# A 2-year longitudinal study of neuropsychological functioning, psychosocial adjustment and rehospitalisation in schizophrenia and major depression

**DOI:** 10.1007/s00406-020-01118-x

**Published:** 2020-04-03

**Authors:** Schaub Annette, Goerigk Stephan, Kim T. Mueser, Hautzinger Martin, Roth Elisabeth, Goldmann Ulrich, Charypar Marketa, Engel Rolf, Möller Hans-Jürgen, Falkai Peter

**Affiliations:** 1 Department of Psychiatry and Psychotherapy, University Hospital, Ludwig Maximilian University of Munich, Nussbaumstr.7, 80336 Munich, Germany; 2 Department of Psychological Methodology and Assesssment, Ludwig Maximilian University of Munich, Leopoldstr. 13, 80802 Munich, Germany; 3 Center for Psychiatric Rehabilitation, Boston University, 940 Commonwealth Avenue, West Boston, MA 02215 USA; 4 Department of Clinical Psychology and Psychotherapy, University of Tübingen, 72026 Tübingen, Germany; 5 Private and Non-Private Praxis, 80333 Munich, Germany; 6 Department of Psychology, Clinical Psychology and Psychotherapy, Ludwig Maximilian University of Munich, Leopoldstrasse 44, 80802 Munich, Germany; 7 Private Praxis, 80336 Munich, Germany; 8 Johannesbad Klinik, 93437 Furth im Wald, Germany

**Keywords:** Schizophrenia, Major depression, Neuropsychological functioning, Psychosocial functioning, Psychoeducation, Cognitive-behavioral therapy, Illness management, Rehospitalisation rate

## Abstract

Neuropsychological functioning turns out to be a rate-limiting factor in psychiatry. However, little is known when comparing neuropsychological and psychosocial functioning in inpatients with schizophrenia or severe depression in their treatment pathways including add-on psychoeducation or the latter combined with cognitive behavioral therapy up to 2-year follow-up. To evaluate this question, we investigated these variables in two randomised controlled trials including 196 patients with DSM-IV schizophrenia and 177 patients with major depression. Outcome measures were assessed in the hospital at pre- and posttreatment and following discharge until 2-year follow-up. We focused on neuropsychological and psychosocial functioning regarding its differences and changes over time in data of two pooled trials. There were significant time effects indicating gains in knowledge about the illness, short and medium-term memory (VLMT) and psychosocial functioning (GAF), however, the latter was the only variable showing a time x study/diagnosis interaction effect at 2-year follow-up, showing significant better outcome in depression compared to schizophrenia. Moderator analysis showed no changes in psychosocial and neuropsychological functioning in schizophrenia and in affective disorders due to age, duration of illness or sex. Looking at the rehospitalisation rates there were no significant differences between both disorders. Both groups treated with psychoeducation or a combination of psychoeducation and CBT improved in neuropsychological and psychosocial functioning as well as knowledge about the illness at 2-year follow-up, however, patients with major depression showed greater gains in psychosocial functioning compared to patients with schizophrenia. Possible implications of these findings were discussed.

**Electronic supplementary material:**

The online version of this article (10.1007/s00406-020-01118-x) contains supplementary material, which is available to authorized users.

## Introduction

Schizophrenia and major depression create a wide range of personal challenges. These cover unpredictable relapses, difficulties with cognitive functioning and loss of social support [1]. According to the World Health Organisation, depression will be the most important contributor to the global burden of disease by 2030 [2]. Prevalence of major depression is around 10% and of schizophrenia around 1% showing a much earlier onset of the illness in schizophrenia compared to depression [3–5].

Psychopharmacological interventions play a major role in the treatment of acute schizophrenia and severe depression. They are to reduce the severity of acute symptoms [6−7] and prevent relapses. However, medications have negligible effects on cognitive and psychosocial functioning [8]. There is need for additional psychosocial interventions to address the 20–30% of patients with schizophrenia having persistent symptoms and relapses despite adherence to antipsychotic medications [6–7] and the 15% of patients with major depression having an unremitting course [9].

Lack of insight and poor treatment adherence with psychopharmacological and psychological interventions are characteristics of both illnesses asking for interventions to increase patients’ understanding of their psychiatric condition, the principles of treatment, and training of strategies to cope with the illness and to pursue goals. Meta-analyses over the last twenty years have shown that cognitive behavioral therapy (CBT) and psychoeducational interventions (PE) are effective in the treatment of schizophrenia and major depression [10–16].

Studies comparing neuropsychological functioning of schizophrenia and major mood disorders have produced somewhat mixed results [17–21]. Looking at neuropsychological functioning in meta-analytic studies, there was a considerable overlap in healthy and depressed persons, however, fewer matching between the latter and persons with schizophrenia [22−25] and major depression with psychotic features (MDDP) [21]. Vulnerability to psychotic experiences conferred a risk for cognitive deficits [23]. Assessment of domain specificity within psychotic disorders, on a backdrop of global impairment, revealed the largest deficit in processing speed in schizophrenia [24] and relatively greater impairment in verbal memory across all groups [20]. There was no evidence for categorical differences between schizophrenia, schizoaffective and affective disorder, however, for a subgroup of patients with schizophrenia having more severe negative symptoms being cognitively more impaired than the rest [26]. Comparing inpatients with schizophrenia, major depression and healthy controls, showed patients with both disorders to be significantly impaired compared to healthy controls and to improve at follow-up [27]. A recent analysis showed effects of psychosocial interventions in outpatient settings on social functioning in depression and schizophrenia [28].

Based on these studies as well as our expertise [29−30], we developed two psychoeducational group programs in schizophrenia and major depression that were combined with coping enhancement in the first and CBT in the latter to be delivered in inpatient treatment including a 2-year follow-up. From our point of view, there was little research on inpatients [31−32], and guidelines [33−36] voting for studies with long-term efficacy needed more approval. The fact that one study focused on schizophrenia and one on major depression would potentially allow interesting comparisons of the clinical and functional outcomes in the two disorders. There are important advantages of this study based on a large sample size and each of the randomized controlled trials based on basic methodology that have been published previously. The paramount aim of this add-on study is therefore to develop more specific and efficient psychological interventions for inpatients with schizophrenia and depression to improve treatment adherence, to minimize depressive symptoms in acute episodes and to prevent relapses and worsening of symptoms.

To our knowledge there are no 2-year follow-up studies available comparing patients with schizophrenia and major depression in different treatment pathways. Psychoeducation [16] and cognitive-behavioral therapy [11−15] have gained prominence in the treatment of schizophrenia and major depression, however, few is known when comparing overall functioning starting with inpatient treatment following a symptom exacerbation up to 2-year outcome. We therefore compare the results of two randomised controlled trials focusing first on long-term effects of 196 inpatients with schizophrenia attending a group-based coping-oriented psychoeducational (COP) [37] or supportive program (SUP). Second, we focus on the long-term effects of 177 depressed inpatients attending an extended clinical management (E-CM), psychoeducational CBT group treatment (PCBT-G) and PCBT combined with individual sessions (PCBT-G + I) [38]. Psychoeducation and CBT were add-on interventions as all patients were treated with pharmacotherapy.

The study focuses on differences and changes in psychoeducational coping-oriented group treatment (COP) in schizophrenia and the combination of psychoeducational CBT group treatment (PCBT-G) in major depression focusing on duration of illness, symptom changes, psychosocial and neuropsychological functioning and, rehospitalisation rates. We expect participants with schizophrenia to have a much earlier onset of the illness [3−5], to worsen over time and to have lower levels of neuropsychological and psychosocial functioning as well as worse course of the illness compared to depressed patients at 2-year outcome [17, 23, 25, 28].

## Methods

### Studies and participants

A total of 196 patients with schizophrenia [37] and 177 with major depression [38] participated at different group programs in randomised controlled trials at the Department of Psychiatry and Psychotherapy, University Hospital, LMU Munich. Inclusion criteria were: (1) 18 to 65 or 69 years old, (2) diagnosis of schizophrenia spectrum disorder or other psychotic disorder respective major depression made by treating psychiatrists according to DSM-IV [39], (3) post-acute stage of the illness (i.e., remission of acute symptoms), (4) proficient in German, (5) sufficiently stable to participate in group therapy, (6) written informed consent. Exclusion criteria were: (1) organic brain syndrome, (2) current drug or alcohol dependence, (3) acute suicidality, (4) level of intelligence < 85. Both studies were approved by the University Institutional Review Board and set up in a comparable time-frame.

The first study compared the long-term effects of a coping-oriented psychoeducational program (COP) with an equally intense supportive therapy (SUP) in schizophrenia. We refer to interventions focusing on education, teaching more effective coping strategies, and cognitive restructuring as “coping-oriented” programs [37, 40–42] as they endeavor to foster more adaptive coping with schizophrenia through a wider variety of strategies than that are usually employed by either cognitive-behavioral or the illness management program. Patients in CBT and in coping-oriented treatment were also offered psychoeducational sessions about the illness and its treatment lasting about 6 weeks in total. The second study compared three treatment conditions including extended clinical management (E-CM), psychoeducational cognitive behavioural group treatment (PCBT-G) or the latter in addition to individual outpatient treatment (PCBT-G + I) in major depression. Detailed information about participants, procedures, organization of the treatment program, its contents and data-analysis is provided [37−38]. There were group treatment guidelines available for both disorders lasting for 12 sessions with two sessions per week to encourage direct communication between inpatients, while also establishing a structure for the therapists to convey essential information and skills. In both studies, the agenda of the sessions were supplemented by materials including flipcharts, handouts and homework assignments [40–42].

### Measures

At baseline and post-treatment, 6–8 weeks later, and 1 and 2 years following discharge from the hospital, we assessed clinical and sociodemographic variables by clinical interviews and questionnaires at both study samples by trained clinical psychologists. This paper comprises both studies focusing on the same criteria of outcome. Psychosocial functioning was assessed with the Global Assessment Functioning Scale (GAF, DSM-IV [39, 43]) ranging from poor (0) to high (100) functioning. Knowledge about schizophrenia or depression and its treatment were assessed using a modified multiple choice test [44]. There were comparable numbers of questions in both instruments with 26 respective 28 questions about diagnosis, its aetiology, symptoms, and treatment strategies in schizophrenia and in depression, however due to a different coding there were only half of the credit points in the first test. Knowledge was defined as number of correct answers, with scores ranging from 0 to 60 in schizophrenia and 0 to 114 in depression. Neuropsychological functioning was assessed with the German version of the Verbal Learning Memory Test [45] measuring verbal learning and memory. It includes immediate recall (reflecting concentration and memory), cumulative learning with exposure and practice, interference, long-delay recall and recognition discriminability. Subjects are instructed to recall as many words of a 15-item list as possible, after each of five separate learning trials. A new list is presented once, requiring immediate or recall of the learning curve, interference, or after a long-delay (20 min) free recall of the first list. Finally, a list of words is read to the subject, who is asked to recognize words from the first list (recognition discriminability). Satisfaction with treatment was assessed using a 4-item questionnaire administered at the end of each treatment group in both studies, covering psychoeducation and practicability of skills learned [46]. At each assessment, information about antipsychotic and anticholinergic medications was obtained, and chlorpromazine-equivalents were calculated [47]. Rehospitalization was determined by a combination of participant interview and review of medical records.

### Data analysis

Baseline differences in demographic and clinical variables as well as in the two samples of patients with schizophrenia or with depression were evaluated with chi-square and independent *t*-tests. For main outcomes, we performed linear mixed-effects regression analyses with three (pre-treatment, 1-year follow-up, 2-year follow-up) measurements for VLMT and four measurements (pre-treatment, post-treatment, 1-year follow-up, 2-year follow-up) for GAF and knowledge, respectively. Measurements were considered as nested within patients, and an unstructured covariance structure was assumed. Time and study/diagnosis as well as their cross-level interaction time × study/diagnosis were introduced as fixed factors, effects of age and duration of disease were controlled for by including these variables as covariates. On the patient level, a random effect was included for the intercept to take individual symptomatic variation at baseline into account. Effect size was reported as Cohen’s *d*. Results were significant at *p* < 0.05. Analyses were performed using the lme4 package in R, version 3.5.2.

## Results

Table 1 summarizes the demographic and clinical data of inpatients with schizophrenia and major depression at pre-treatment.

**Table 1:** Clinical and sociodemographic variables at both study samples at baseline, post-treatment and 1-year to 2-year follow-up

Patients with schizophrenia (*n* = 196)									Patients with depression (*n* = 177)								
Age *M* = 33.6 (SD = 11.3) years old									*M* = 47.89 (SD = 12.6) years old					*t* = 11.55, *p* > .01			
47% female									57% female								
71% living alone									39% living alone								
Duration of illness 6.2 years (SD = 7.6)									9.2 years (SD = 10.09)					*t* = − 3.22, *p* > .01			
	Baseline		Post-treatment		1-year		2-year			Baseline		Post-treatment		1-year		2-year	
	*M*	SD	*M*	SD	*M*	SD	*M*	SD		*M*	SD	*M*	SD	*M*	SD	*M*	SD
*GAF scores in schizophrenia*									*GAF Scores in depression*								
COP	49.48	14.41	61.28	13.9	73.00	20.62	74.76	16.30	E-CM	49.07	13.66	78.89	3.09	84.86	10.49	82.65	10.33
SUP	48.48	12.40	58.60	13.28	66.37	19.90	71.89	20.00	PCBT-G	53.72	12.71	71.58	14.10	83.93	10.09	82.95	11.13
									PCBT-G + I	52.27	11.69	71.04	13.10	80.11	11.32	82.93	8.72
*VLMT* [45] *Scores in schizophrenia*									*VLMT scores in depression*								
VLMT*	6.34	2.36		7.59	2.66	8.36	5.92		VLMT*	6.37				7.35		8.21	
VLMT**	47.58	12.52		53.57	11.88	54.90	10.10		VLMT**	50.86	9.64			54.68	11.20	56.48	9.23

*GAF* global assessment functioning scale, *E-CM* extended clinical management, *COP* coping-oriented treatment (= BOT), *PCBT-G* psychoeducational cognitive behavioral treatment-group (= VOG), *SUP* supportive treatment, *PCBT-G* + *I* PCBT-G + individual treatment, VLMT* verbal learning memory test, immediate recall, VLMT** verbal learning memory test, learning curve

This table shows the number of hospitalizations over the 2 years and mainly focuses on participants in COP in study 1 and for PCBT in study 2. Patients with schizophrenia participating at the coping-oriented study (COP) were significantly younger compared to patients with depression attending the psychoeducational cognitive behavioral treatment group (PCBT-G). The first sample also had a significantly shorter duration of illness. Age and duration of illness were included as covariates in the following data analysis. There were no significant differences in sex between the two groups with 53% male in the first vs. 57% female in the second sample: chi^2^ test: Pearson’s Chi-squared test with Yates’ continuity correction *X*^2^ (1) = 2.6979, *p* = 0.1005. However, 71% of patients with schizophrenia were single compared to 39% of depressed patients and 61% of patients with depression had a relationship or were married (*t* = 11.55, *p* > 0.01). The pre-treatment scores of GAF [39] and VLMT [41] in both samples were comparably low *M* = 49.48 (SD = 14.41), *M* = 49.07 (SD = 13.66) indicating symptoms at a moderate level including flat affect, circumstantial speech, occasional panic attacks or moderate difficulty in social and occupational functioning e.g., few friends, conflicts with peers or co-workers.

**Fig. 1:**
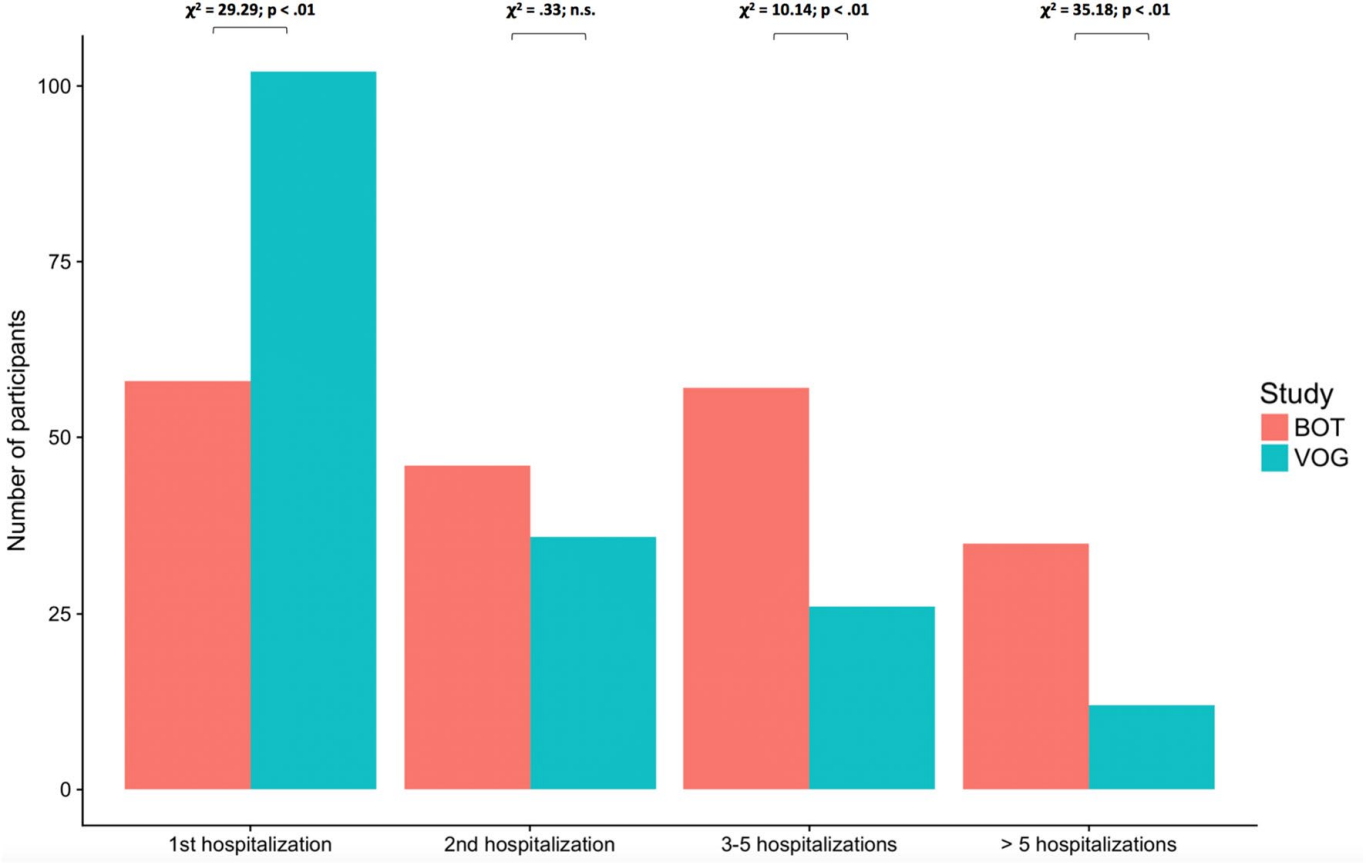
Number of hospitalisations in COP (BOT) vs number of hospitalisations in P-CBT (VOG*)* at pre-assessment

Figure 1 shows the number of hospitalizations at pre-assessment in both samples. 45% of patients with depression versus 30% of patients with schizophrenia had their first episode and hospitalization (*x*^2^ = 29.29, *p* < 0.01), however, in schizophrenia there was an increasing number of hospitalizations indicating a more chronic course of the illness.

Table 2 shows neuropsychological and psychosocial functioning from pre-treatment to 2-year follow-up. The average knowledge [^44^] about schizophrenia and its treatment at pre-treatment was *M* = 76.54; SD = 12.45 compared to *M* = 92.85; SD = 12.51 in depression. At 2-year follow-up both groups increased their knowledge with an average knowledge about schizophrenia and its treatment with *M* = 85.57; SD = 12.33 compared to *M* = 99.48; SD = 11.93 in depression (*p* < 001). However, there was no significant time × study effect of knowledge. The average scores of the Verbal Memory Test [^45^] indicating immediate recall and short-term memory were comparable at pre-assessment in COP and PCBT with 6.43 and 6.37. At 2-year follow-up, the average score in COP was *M* = 8.36 (SD = 2.92) and in CBT-G, *M* = 8.21 (SD = 2.2) showing significant time effects for both groups (*p* =  < 0.001), however, no time × interaction effect. Looking at the learning curves in medium-memory [^45^], there were also significant time effects in schizophrenia and depression (*p* =  < 0.001) as well as a study effect (*p* = 0.012). The first group showed a broader deviation of scores than the latter. The GAF scores [^39^] of both studies showed comparable mean scores in schizophrenia and in depression at pre-treatment, however, an increasing gap during the course of the study at 2-year follow-up showed a significant time-effect as well as time × treatment effect in favour of major depression (*p* =  < 0.001). There was no difference in satisfaction with the treatment program in both studies as we adapted the treatment strategies to the given possibilities of the patients [^46^].

**Table 2:** Neuropsychological and psychosocial functioning from pre-treatment to 2-year follow-up

	Intercept			Time			Study			Time x study			Model fit
Measure	*β*	(95%CI)	*P*	*β*	(95%CI)	*P*	*β*	(95%CI)	*P*	*β*	(95%CI)	*P*	*R*^2^/Ω_0_^2^
Knowledge	77.46	75.79–79.12	< .001	2.54	1.68–3.41	< .001	16.42	14.48–18.36	< .001	− 0.49	− 1.80–0.81	.459	.608 / .598
VLMT item 1	6.42	6.07–6.76	< .001	0.94	0.71–1.17	< .001	− 0.06	− 0.55–0.44	.815	− 0.00	− 0.36–0.36	.995	.734 / .678
VLMT 1–5	47.82	46.14–49.49	< .001	3.02	2.06–3.98	< .001	3.06	0.68–5.45	.012	0.35	− 1.15–1.85	.648	.823 / .787
GAF	50.02	48.09–51.95	< .001	8.56	7.59–9.53	< .001	4.69	1.90–7.49	.001	2.85	1.38–4.31	< .001	.677 / .663

Estimate, regression weight; 95%CI, confidence interval; *R*^2^/Ω_0_^2^, variance explained by model; Model uses baseline as reference measurement for fixed model coefficients and COP as study reference

We found significant time effects for GAF (*F*(1,796.47) = 683.067, *p* < 0.001), VLMT immediate recall (*F*(1,416.87) = 90.50, *p* < 0.001), VLMT learning curve (*F*(1,354.57) = 61.83, *p* < 0.001), and knowledge (*F*(1,799.42) = 40.37, *p* < 0.001), indicating group-unspecific improvements over time in all outcomes. Significant group effects for GAF (*F*(4,688.07) = 3.89, *p* = 0.004), VLMT immediate recall (*F*(4,488.10) = 3.71, *p* = 0.006), VLMT learning curve (*F*(4,420.51) = 6.867, *p* < 0.001), and knowledge (*F*(4,891.99) = 74.97, *p* < 0.001), are reflective of a generally higher level of functioning in the depressed patients as compared to the schizophrenic sample. For the GAF, a significant time × group interaction was found (*F*(4,791.82) = 4.47, *p* = 0.002), revealing group-specific differences in trajectories between E-CM and COP, E-CM and SUP, PCBT-G and SUP, as well as PCBT-G + I and SUP (Table 2).

Data about the course of the illness in both studies are shown in Table 3: Flow of participants and cumulative rehospitalisation rates at 2-year follow-up in both studies. There was a higher treatment adherence in schizophrenia [37] (125 of 196) compared to major depression [38] (96 of 177). Although we expected the coping-oriented treatment program to be superior to supportive treatment in preventing rehospitalization at 2-year follow-up period, both groups had comparable but relatively low rates of rehospitalization over this period (COP: 38, SUP: 37) [37]. These results are similar to other studies [48−49], such as the recently for the National In stitute of Mental Health sponsored Recovery After Initial Schizophrenia Episode-Early Treatment Program (RAISE-ETP) study [48], a cluster randomized controlled trial involving 34 sites in which the 2-year hospitalization rate for participants in the specialty comprehensive treatment program for first episode psychosis was 34%, compared to 37% for those who received usual community care. The psychoeducational approach in the PIP-Study offering information about symptoms, pharmacotherapy and relapse plan to patients and their relatives in distinct groups was 41% compared to 58% in standard treatment [49]. The inclusion of relatives was mandatory in this design, and thus the results could reflect the effects of family psychoeducation, an established practice for reducing relapses and hospitalizations in schizophrenia [44]. This study included mainly affiliated outpatients, whereas in our sample only 15% of the patients were temporary outpatients. Comparable to this study our participants were inpatients, however, our inclusion criteria were broader as we also included first episode patients (about 1/3 of the sample) and there was no request for study participation that relatives also attended psychoeducational groups. At 2-year follow up, participants in PCBT-G [38] had significantly lower rehospitalisation rates (27%) than those in E-CM (40%) (*t* (98.61) = 2.96; *p* ≤ 0.004; NNT = 3.8) as well as those in CBT-G + I (34%) (*t* (114.01) = 2.22; *p* ≤ 0.028; NNT = 4.7), whereas the rehospitalisation rates between PCBT-G + I and E-CM did not differ significantly. Table 3: Flow of participants and cumulative rehospitalisation rates at 2-year follow-up in both studies.

**Table 3:** Flow of participants and cumulative rehospitalisation rates at 2-year follow-up in both studies

Schizophrenia (COP-study)		Major depression (PCBT-G-study)		
Randomised: 198		Randomized: 177		
Conditions				
COP	SUP	E-CM	PCBT-G	PCBT-G + I
*Pre-treatment*				
*n* = 100	*n* = 96	*n* = 58	*n* = 59	*n* = 60
*Analysed at 1-year follow-up*				
*n* = 66	*n* = 64	*n* = 27	*n* = 23	*n* = 20
*Analysed at 2-year follow-up*				
*n* = 70	*n* = 55	*n* = 31	*n* = 30	*n* = 25
*Rehospitalisation rates*				
38%	37%	40%*	27%*	34%

*COP* coping-oriented treatment, *SUP* supportive treatment, *PCBT-G* cognitive-behavioral group therapy for inpatients, *PCBT-G* + *I* cognitive behavioral group for inpatients and individual therapy for outpatients, *E-CM* extended clinical management for inpatients

^*^p = .05

See also Supplementary data: (Supplementary Fig. 1, Supplementary Tables 1, 2, 3).

## Discussion

Comparable to other studies including patients with MD and with schizophrenia, the first group was significantly older at illness onset, had a shorter duration of illness and was socially better integrated than patients with schizophrenia—although in our sample there was no majority of women [3–5]. Our hypothesis about neuropsychological and psychosocial functioning in schizophrenia was confirmed in psychosocial functioning, however, rejected in neuropsychological functioning. There were no interaction effects in knowledge about the illness [43], in immediate recall and the learning curve of verbal learning [45], however, in global functioning [39], there were higher scores in depression compared to schizophrenia at 2-year follow-up. In our study pre-scores of GAF [39] were at a low-level comparable in schizophrenia and in depression, however, the gap increased at 2-year follow-up (*p* =  < 0.001) showing time × treatment effects in favour of MD as well as main effects. Patients with schizophrenia were rated as having symptoms, transient, and expectable reactions to psychosocial stressors, having only slight impairment in social, occupational or school functioning compared to patients with depression who were rated as having no or minimal symptoms, good functioning in all areas, interested and involved in a wide range of activities. Patients with depression were rated as socially effective, generally satisfied with life and having no more than everyday problems or concerns. Comparable to other studies psychosocial interventions for depressed patients [28] were effective at improving social functioning, however, in schizophrenia, evidence from high quality trials is limited.

Inpatients with schizophrenia performed lower than patients with depression at pre-assessments in some tests, however, better in others and at follow-up, both patient groups showed improved performance. The findings do not show greater cognitive impairment in schizophrenia compared to depression to be a trait marker and differentiating both disorders at the basis of cognitive functioning seems to be less specific than expected. [27] The only significant study/diagnosis × time interaction is in psychosocial functioning showing patients with schizophrenia to be more impaired at 2-year follow-up compared to patients with depression. In both studies the number of participants decreased at 2-year follow-up, however, there was a higher treatment adherence in schizophrenia (125 of 196) compared to major depression (96 of 177) shown in Table 3: Flow of participants and cumulative rehospitalisation rates at 2-year follow-up in both studies [37–38].

For a long time, investigating and comparing patients with schizophrenia and major depression was considered useless as it was like “comparing apples with peaches”. However, despite this critique and encouraged by recent therapeutic trends in psychoeducation [49−50] and meta-cognitive training [51−53] as well as the results of our working group on schizophrenia and major depression presented at the 6th Kraepelin Symposium last year [54], we analysed the data of two randomised studies on this topic. A Consensus Cognitive Battery of cognitive deficits (MATRICS) were set up including speed of processing, attention/vigilance, working memory, verbal and visual learning as well as reasoning and problem solving [55]. This was the basis for modifications in treatment models based on current research to improve cognition in schizophrenia. Aerobic exercise and cognitive training may have synergistic effects on learning and overall cognitive functioning that can enhance the impact of cognitive training alone [56−57].

The results of our studies are in line with other psychoeducational and cognitive-behavioral interventions in controlled studies in schizophrenia and in major depression. The large number of patients and the fact that all groups in both studies were comparable regarding clinical and demographic data including chlorpromazine equivalents as well as treatment time involved in therapy are the strengths of the study. There was a moderate drop-out rate that is comparable with other studies.

Limitations of these studies are that we did not biologically analyze the level of medication to indicate treatment adherence. All treatment groups were established in the same setting and thus, we could not control interactions between patients and we could not guarantee the blindness of the raters throughout the studies. The number of hospitalizations refers to a vague construct as it might also be dependent on the social situation or illness management and less on psychopathological criteria. A higher rate in psychopathological assessments could have optimized both studies.

During the last 20 years, illness management programs covering primary, secondary and tertiary prevention gained importance in psychiatry. Important goals are to prevent the onset of the illness, its reoccurrence of symptoms and to improve quality of life by reducing disability and limiting or delaying recurrence in those already affected by a disease. There is a need to develop a concept of the self not exclusively defined in terms of the illness but based on a conception of one’s individuality and on the remaining possibilities of life [29–30].

What are the beneficial psychotherapeutic factors of COP and PCBT-G? We agree with Grawe [58] and colleagues who listed training competencies, providing insight and interactive processes [59] as well as high structure and transparency. We would also add sharing experiences, having the possibility to increase social skills and gaining social buffer in coping with the illness and anti-stigma processes. [60] Identifying and managing early warning signs of relapse, developing efficient coping strategies as well as training skills to cope with the stressors are very important in the treatment of schizophrenia [37−38, 48–51].

In the last 20 years, there was a great change in psychotherapy and we are optimistic to reduce even more prejudice in psychiatry in the following years [59, 60]. 43 years ago, postpsychotic depression in schizophrenia was seen as a relatively neglected area despite the risk of suicide and prolonged suffering [61]. Nowadays it looks that this topic is still relatively neglected although it seems natural that it would occur sometimes [62]. Interest shifted to depression mediating cognition impairment in schizophrenia or to the possible similarity of depressive and schizophrenia anhedonia. From our point of view, it looks interesting to tackle the role of depression in the affective/non-affective psychosis dichotomy.

## Conclusions

The results of both studies are encouraging because they suggest that the effects of a relatively time-limited, inpatient COP or PCBT-G were sustained over a 2-year follow-up period after patients had been discharged into the community, and they support the potential benefits of psychosocial treatment during the inpatient phase. The study was conducted in a treatment setting that provided a broad range of psychotherapeutic and rehabilitative interventions in addition to pharmacological treatment, and therefore, one might expect better overall outcomes for participants in both groups compared to a treatment setting more narrowly focused on pharmacological stabilization and safety.

In summary, we found that both patients with schizophrenia and affective disorders improved their symptoms over the course of the illness. Both group interventions (COP and PCBT-G) led to greater increases in knowledge about mental illness. There was an increase of psychosocial functioning significantly higher mainly in favour of major depression. Global Assessment functioning, knowledge about the illness, verbal learning memory test improved more pronounced in the depressed patients compared to patients with schizophrenia. The benefits of the program on symptoms were sustained over two years following discharge from the hospital. There were benefits in group therapy COP and PCBT even at 2-year follow-up, however, the effects were more pronounced in patients with major depression.

Research on associations between psychotic symptoms and cognitive impairment, differential developmental mechanisms across affective and non-affective psychosis, and treatment options for cognitive impairment are important steps for improving the lives of individuals with psychosis.

## Electronic supplementary material

Below is the link to the electronic supplementary material.

Supplementary file1 (DOCX 147 kb)
