# Supplementary material for: A 2-year longitudinal study of neuropsychological functioning, psychosocial adjustment and rehospitalisation in schizophrenia and major depression
Source: Eur Arch Psychiatry Clin Neurosci. 2020 Apr 3;270(6):699–708. doi: 10.1007/s00406-020-01118-x (PMC7423783; doi:10.1007/s00406-020-01118-x)
Supplement: Supplementary file 1 — Supplementary file1 (DOCX 147 kb) [file 406_2020_1118_MOESM1_ESM.docx]

Electronic supplement

**A two-year longitudinal study of neuropsychological functioning, psychosocial adjustment and rehospitalisation in schizophrenia and major depression**.

Schaub Annette*^1^, Goerigk Stephan^1,2^, Mueser Kim T^3^, Hautzinger Martin^4^, Roth Elisabeth^5^, Goldmann Ulrich^6,7^, Charypar Marketa^8^, Engel Rolf^1^, Möller Hans-Jürgen^1^, Falkai Peter^1^

^1^Department of Psychiatry and Psychotherapy, University Hospital, Ludwig Maximilian University of Munich, Nussbaumstr.7, D - 80336 Munich*

^2^Department of Psychological Methodology and Assesssment, Leopoldstr. 13, Ludwig Maximilian University of Munich, D – 80802 Munich

^3^Center for Psychiatric Rehabilitation, Boston University, 940 Commonwealth Avenue, West Boston, MA 02215, U.S.A.

^4^Department of Clinical Psychology and Psychotherapy, University of Tübingen, D - 72026 Tübingen

^5^Private and non-private praxis, D – 80333 Munich, Germany

^6^Department of Psychology, Clinical Psychology and Psychotherapy, Ludwig Maximilian University of Munich Leopoldstrasse 44, 80802 München

^7^Private praxis, D-80336 Munich

^8^Johannesbad Klinik, D-93437 Furth im Wald

*Corresponding author:

Dr. Annette Schaub,

Department of Psychiatry and Psychotherapy. University Hospital, LMU Munich,

D- 80336 Munich,

Nussbaumstraße 7,

Germany;

Phone: 0049-89-440052779,

Fax: 0049-89-440052729,

e-mail: annette.schaub@med.uni-muenchen.de

**Supplementary Figure 1: Group-specific change in global functioning and cognitive outcomes**


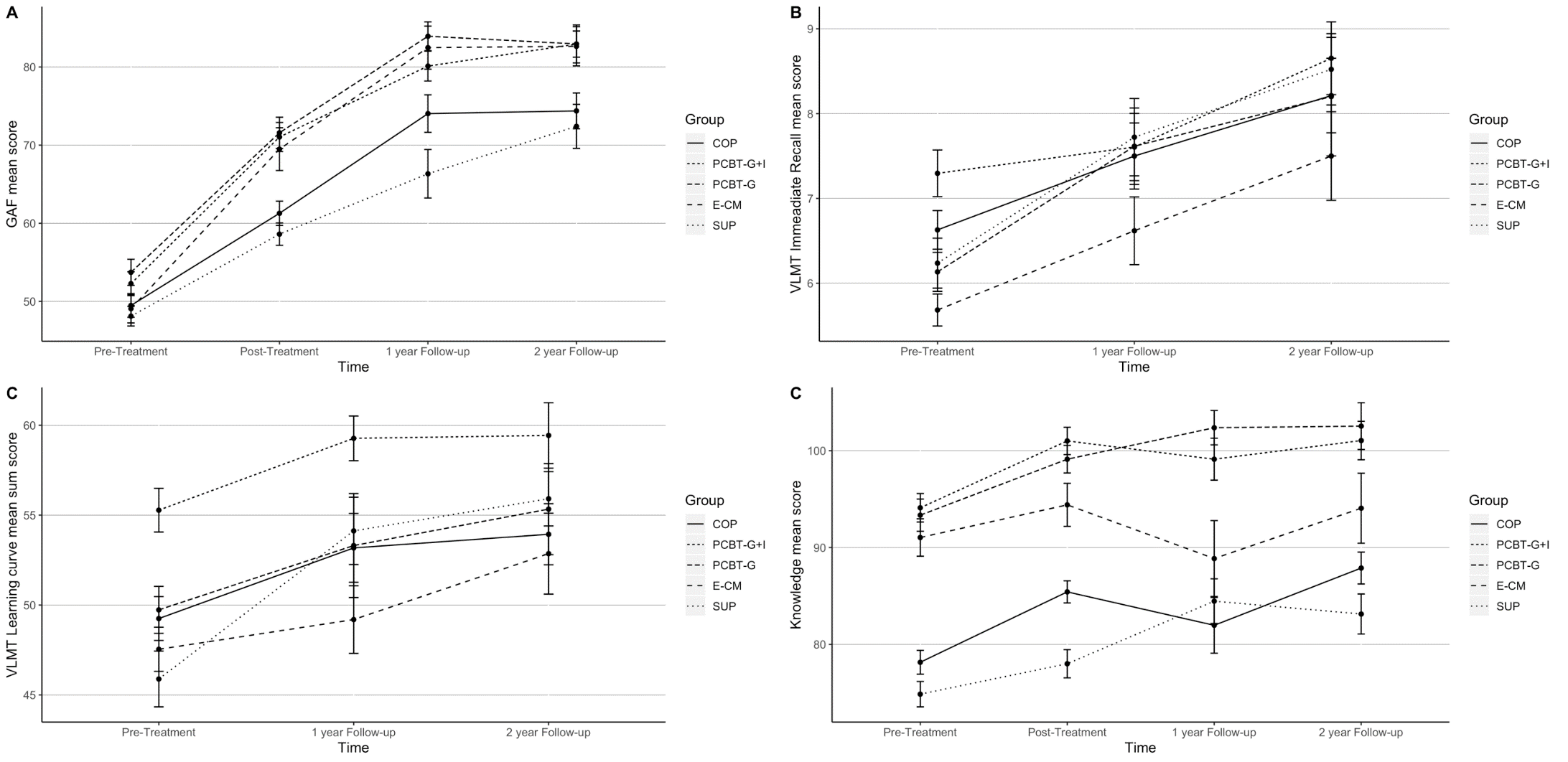


**Supplementary Table 1**

**Group-specific change in outcome measures**

| **Study** | **Group** | **Measure** | **Baseline** | | **Post-Treatment** | | **1 Year** | | **2 Year** | |
| --- | --- | --- | --- | --- | --- | --- | --- | --- | --- | --- |
|  |  |  | **M** | **SD** | **M** | **SD** | **M** | **SD** | **M** | **SD** |
| P-CBT | E-CM | GAF | 49.07 | 13.66 | 69.49 | 16.67 | 82.48 | 12.62 | 82.65 | 10.33 |
|  |  | VLMT Immediate recall | 5.69 | 1.38 |  |  | 6.62 | 1.83 | 7.50 | 1.95 |
|  |  | VLMT Learning curve | 47.54 | 9.03 |  |  | 49.19 | 8.61 | 52.86 | 8.42 |
|  |  | Knowledge Test | 91.04 | 14.17 | 94.41 | 13.49 | 88.86 | 18.36 | 94.06 | 14.87 |
|  | PCBT-G | GAF | 53.72 | 12.71 | 71.58 | 14.10 | 83.93 | 10.09 | 82.95 | 11.13 |
|  |  | VLMT Immediate recall | 6.13 | 1.66 |  |  | 7.62 | 2.30 | 8.20 | 2.70 |
|  |  | VLMT Learning curve | 49.73 | 9.41 |  |  | 53.31 | 14.74 | 55.33 | 9.81 |
|  |  | Knowledge Test | 93.34 | 12.13 | 99.11 | 9.43 | 102.36 | 8.31 | 102.53 | 9.96 |
|  | PCBT-G+I | GAF | 52.27 | 11.69 | 71.04 | 13.10 | 80.11 | 11.32 | 82.93 | 8.72 |
|  |  | VLMT Immediate recall | 7.30 | 2.02 |  |  | 7.61 | 2.28 | 8.65 | 2.06 |
|  |  | VLMT Learning curve | 55.28 | 8.94 |  |  | 59.27 | 7.14 | 59.43 | 8.73 |
|  |  | Knowledge Test | 94.10 | 11.16 | 101.00 | 9.47 | 99.12 | 11.02 | 101.04 | 10.10 |
| COP | COP | GAF | 49.48 | 14.41 | 61.27 | 13.89 | 74.03 | 18.27 | 74.38 | 17.00 |
|  |  | VLMT Immediate recall | 6.63 | 2.05 |  |  | 7.50 | 2.64 | 8.21 | 3.01 |
|  |  | VLMT Learning curve | 49.25 | 10.99 |  |  | 53.18 | 12.83 | 53.94 | 11.63 |
|  |  | Knowledge Test | 78.16 | 12.08 | 85.43 | 10.79 | 81.98 | 19.29 | 87.89 | 10.89 |
|  | SUP | GAF | 48.11 | 11.94 | 58.60 | 13.28 | 66.33 | 20.12 | 72.40 | 19.30 |
|  |  | VLMT Immediate recall | 6.24 | 2.63 |  |  | 7.72 | 2.73 | 8.52 | 2.85 |
|  |  | VLMT Learning curve | 45.89 | 13.78 |  |  | 54.12 | 10.59 | 55.91 | 10.13 |
|  |  | Knowledge Test | 74.87 | 12.68 | 78.01 | 13.19 | 84.47 | 13.02 | 83.14 | 13.38 |

Note: E-CM Extended Clinical Management; PCBT-G Psychoeducational Cognitive Behavioral Treatment- Group; PCBT-G+I PCBT-G+Individual Treatment; COP Coping-oriented Treatment; SU Supportive Treatment; GAF Global Assessment Functioning Scale; VLMT Verbal Learning Memory Test

**Supplementary Table 2: Group differences in trajectories of global functioning and cognition**

|  | **GAF** | | | **VLMT Immediate recall** | | | **VLMT Learning curve** | | | **Knowledge** | | |
| --- | --- | --- | --- | --- | --- | --- | --- | --- | --- | --- | --- | --- |
| **Group** | **∆ Slope** | **P** | **d** | **∆ Slope** | **P** | **d** | **∆ Slope** | **P** | **d** | **∆ Slope** | **P** | **d** |
| E-CM vs PCBT-G | -2.08 | 0.151 | -0.47 | 0.33 | 0.375 | 0.43 | 1.02 | 0.506 | 0.22 | 2.77 | **0.029*** | 0.63 |
| E-CM vs PCBT-G+I | -2.06 | 0.141 | -0.49 | -0.20 | 0.568 | -0.21 | 0.09 | 0.950 | 0.02 | 1.23 | 0.300 | 0.29 |
| E-CM vs COP | -3.95 | **0.002**** | -0.84 | -0.12 | 0.707 | -0.13 | -1.08 | 0.403 | -0.21 | 1.76 | 0.105 | 0.37 |
| E-CM vs SUP | -4.79 | **<.001***** | -1.14 | 0.23 | 0.464 | 0.21 | 1.50 | 0.251 | 0.25 | 1.91 | 0.080 | 0.37 |
| PCBT-G vs PCBT-G+I | 0.02 | 0.987 | 0.01 | -0.53 | 0.120 | -0.55 | -0.93 | 0.504 | -0.19 | -1.54 | 0.198 | -0.40 |
| PCBT-G vs COP | -1.86 | 0.118 | -0.40 | -0.45 | 0.145 | -0.47 | -2.10 | 0.096 | -0.41 | -1.01 | 0.353 | -0.21 |
| PCBT-G vs SUP | -2.70 | **0.026*** | -0.65 | -0.10 | 0.751 | -0.09 | 0.48 | 0.705 | 0.08 | -0.85 | 0.439 | -0.17 |
| PCBT-G+I vs COP | -1.89 | 0.095 | -0.42 | 0.08 | 0.771 | 0.08 | -1.17 | 0.295 | -0.22 | 0.53 | 0.592 | 0.11 |
| PCBT-G+I vs SUP | -2.72 | **0.018*** | -0.68 | 0.43 | 0.121 | 0.35 | 1.41 | 0.214 | 0.22 | 0.68 | 0.495 | 0.13 |
| COP vs SUP | -0.84 | 0.395 | -0.19 | 0.35 | 0.139 | 0.30 | 2.59 | **0.008**** | 0.41 | 0.15 | 0.861 | 0.04 |

Note: ∆ mean difference in group slopes (i.e. adjusted change per measurement); d effect size Cohens d; E-CM Extended Clinical Management; PCBT-G Psychoeducational Cognitive Behavioral Treatment- Group; PCBT-G+I PCBT-G+Individual Treatment; COP Coping-oriented Treatment; SU Supportive Treatment; GAF Global Assessment Functioning Scale; VLMT Verbal Learning Memory Test

**Supplementary Table 3**: **Analysis of moderators for covariates in COP and PCBT studies showing no change in outcome moderated by age or gender (ES = experimental subject)**

**Anova**(**lmer**(GAF**~**TIME*****Gender**+**(TIME**|ES**), data = COP_gaf_long))

## Type III Analysis of Variance Table with Satterthwaite's method
## Sum Sq Mean Sq NumDF DenDF F value Pr(>F)
## TIME 25120.8 25120.8 1 141.15 202.3492 < 2.2e-16 ***
## Gender 962.9 962.9 1 183.73 7.7562 0.005914 **
## TIME:Gender 10.8 10.8 1 141.15 0.0874 0.767981
## Signif. c odes: 0 '***' 0.001 '**' 0.01 '*' 0.05 '.' 0.1 ' ' 1

**Anova**(**lmer**(GAF**~**TIME*****Gender**+**(TIME**|ES**), data = PCBT_gaf_long))

## Type III Analysis of Variance Table with Satterthwaite's method
## Sum Sq Mean Sq NumDF DenDF F value Pr(>F)
## TIME 57014 57014 1 361.72 466.9358 < 2e-16 ***
## Gender 777 777 1 180.52 6.3627 0.01252 *
## TIME:Gender 113 113 1 361.72 0.9260 0.33654
## Signif. codes: 0 '***' 0.001 '**' 0.01 '*' 0.05 '.' 0.1 ' ' 1

**Anova**(**lmer**(GAF**~**TIME*****Age**+**(TIME**|**ES), data = COP_gaf_long))

## Type III Analysis of Variance Table with Satterthwaite's method
## Sum Sq Mean Sq NumDF DenDF F value Pr(>F)
## TIME 4860.8 4860.8 1 136.30 39.1010 4.837e-09 ***
## Age 318.5 318.5 1 181.28 2.5618 0.11121
## TIME:Age 348.9 348.9 1 135.22 2.8068 0.09618 .
## Signif. codes: 0 '***' 0.001 '**' 0.01 '*' 0.05 '.' 0.1 ' ' 1

**Anova**(**lmer**(GAF**~**TIME*****Age**+**(TIME**|**ES), data = PCBT_gaf_long))

## Type III Analysis of Variance Table with Satterthwaite's method
## Sum Sq Mean Sq NumDF DenDF F value Pr(>F)
## TIME 2877.83 2877.83 1 365.18 23.5468 1.809e-06 ***
## Age 0.00 0.00 1 181.68 0.0000 0.9957
## TIME:Age 23.45 23.45 1 365.92 0.1918 0.6616
## Signif. codes: 0 '***' 0.001 '**' 0.01 '*' 0.05 '.' 0.1 ' ' 1

**Anova**(**lmer**(knowledge**~**TIME*****Gender**+**(TIME**|**ES), data = knowledge COP_long))

## Type III Analysis of Variance Table with Satterthwaite's method
## Sum Sq Mean Sq NumDF DenDF F value Pr(>F)
## TIME 2484.27 2484.27 1 94.072 33.4949 9.348e-08 ***
## Gender 88.06 88.06 1 191.069 1.1874 0.2772
## TIME:Gender 1.51 1.51 1 94.072 0.0204 0.8867
## Signif. codes: 0 '***' 0.001 '**' 0.01 '*' 0.05 '.' 0.1 ' ' 1

**Anova**(**lmer**(knowledge**~**TIME*****Gender**+**(TIME**|**ES), data = knowledge PCBT_long))

## Type III Analysis of Variance Table with Satterthwaite's method
## Sum Sq Mean Sq NumDF DenDF F value Pr(>F)
## TIME 810.67 810.67 1 95.384 29.1695 4.855e-07 ***
## Gender 0.07 0.07 1 167.106 0.0024 0.9607
## TIME:Gender 44.53 44.53 1 95.384 1.6024 0.2087
## Signif. codes: 0 '***' 0.001 '**' 0.01 '*' 0.05 '.' 0.1 ' ' 1

**Anova**(**lmer**(knowledge**~**TIME*****Age**+**(TIME**|**ES), data = knowledge COP_long))

## Type III Analysis of Variance Table with Satterthwaite's method
## Sum Sq Mean Sq NumDF DenDF F value Pr(>F)
## TIME 313.26 313.26 1 92.237 4.2273 0.04260 *
## Age 290.65 290.65 1 192.846 3.9222 0.04907 *
## TIME:Age 3.17 3.17 1 90.413 0.0428 0.83661
## Signif. codes: 0 '***' 0.001 '**' 0.01 '*' 0.05 '.' 0.1 ' ' 1

**Anova**(**lmer**(knowledge**~**TIME*****Age**+**(TIME**|**ES), data = knowledge PCBT_long))

## Type III Analysis of Variance Table with Satterthwaite's method
## Sum Sq Mean Sq NumDF DenDF F value Pr(>F)
## TIME 185.16 185.16 1 109.71 6.7235 0.01081 *
## Age 1139.45 1139.45 1 167.27 41.3755 1.267e-09 ***
## TIME:Age 39.95 39.95 1 105.39 1.4506 0.23113
## Signif. codes: 0 '***' 0.001 '**' 0.01 '*' 0.05 '.' 0.1 ' ' 1

**Anova**(**lmer**(vlmt1**~**TIME*****Gender**+**(1**|ES**), data = COP_vlmt1_long))

## Type III Analysis of Variance Table with Satterthwaite's method
## Sum Sq Mean Sq NumDF DenDF F value Pr(>F)
## TIME 177.112 177.112 1 218.40 51.8265 9.642e-12 ***
## Gender 1.660 1.660 1 253.57 0.4857 0.4865
## TIME:Gender 0.534 0.534 1 218.40 0.1561 0.6931
## Signif. codes: 0 '***' 0.001 '**' 0.01 '*' 0.05 '.' 0.1 ' ' 1

**Anova**(**lmer**(vlmt1**~**TIME*****Gender**+**(1**|**ES), data = PCBT_vlmt1_long))

## Type III Analysis of Variance Table with Satterthwaite's method
## Sum Sq Mean Sq NumDF DenDF F value Pr(>F)
## TIME 108.609 108.609 1 195.73 48.8437 4.249e-11 ***
## Gender 3.435 3.435 1 235.76 1.5447 0.2152
## TIME:Gender 0.878 0.878 1 195.73 0.3951 0.5304
## Signif. codes: 0 '***' 0.001 '**' 0.01 '*' 0.05 '.' 0.1 ' ' 1

**Anova**(**lmer**(vlmt1**~**TIME*****Age**+**(1**|**ES), data = COP_vlmt1_long))

## Type III Analysis of Variance Table with Satterthwaite's method
## Sum Sq Mean Sq NumDF DenDF F value Pr(>F)
## TIME 27.2794 27.2794 1 215.87 7.9773 0.005181 **
## Age 5.5333 5.5333 1 254.22 1.6181 0.204521
## TIME:Age 1.0198 1.0198 1 217.41 0.2982 0.585563
## Signif. codes: 0 '***' 0.001 '**' 0.01 '*' 0.05 '.' 0.1 ' ' 1

**Anova**(**lmer**(vlmt1**~**TIME*****Age**+**(1**|**ES), data = PCBT_vlmt1_long))

## Type III Analysis of Variance Table with Satterthwaite's method
## Sum Sq Mean Sq NumDF DenDF F value Pr(>F)
## TIME 44.883 44.883 1 210.82 20.1210 1.194e-05 ***
## Age 33.370 33.370 1 247.20 14.9601 0.0001405 ***
## TIME:Age 16.278 16.278 1 209.05 7.2976 0.0074712 **
## Signif. codes: 0 '***' 0.001 '**' 0.01 '*' 0.05 '.' 0.1 ' ' 1

**Aanova**(**lmer**(vlmt1to5**~**TIME*****Gender**+**(1**|**ES), data = COP_vlmt1to5_long))

## Type III Analysis of Variance Table with Satterthwaite's method
## Sum Sq Mean Sq NumDF DenDF F value Pr(>F)
## TIME 1574.82 1574.82 1 183.79 35.6467 1.196e-08 ***
## Gender 5.55 5.55 1 209.32 0.1257 0.7232
## TIME:Gender 94.43 94.43 1 183.79 2.1374 0.1455
## Signif. codes: 0 '***' 0.001 '**' 0.01 '*' 0.05 '.' 0.1 ' ' 1

**Anova**(**lmer**(vlmt1to5**~**TIME*****Gender**+**(1**|**ES), data = PCBT_vlmt1to5_long))

## Type III Analysis of Variance Table with Satterthwaite's method
## Sum Sq Mean Sq NumDF DenDF F value Pr(>F)
## TIME 1536.30 1536.30 1 177.08 32.4555 4.996e-08 ***
## Gender 306.22 306.22 1 217.43 6.4691 0.01167 *
## TIME:Gender 50.10 50.10 1 177.08 1.0585 0.30497
## Signif. codes: 0 '***' 0.001 '**' 0.01 '*' 0.05 '.' 0.1 ' ' 1

**Anova**(**lmer**(vlmt1to5**~**TIME*****Age**+**(1**|**ES), data = COP_vlmt1to5_long))

## Type III Analysis of Variance Table with Satterthwaite's method
## Sum Sq Mean Sq NumDF DenDF F value Pr(>F)
## TIME 437.95 437.95 1 181.64 9.9070 0.001925 **
## Age 32.33 32.33 1 209.80 0.7314 0.393400
## TIME:Age 73.50 73.50 1 182.82 1.6626 0.198882
## Signif. codes: 0 '***' 0.001 '**' 0.01 '*' 0.05 '.' 0.1 ' ' 1

**Anova**(**lmer**(vlmt1to5**~**TIME*****Age**+**(1**|**ES), data = PCBT_vlmt1to5_long))

## Type III Analysis of Variance Table with Satterthwaite's method
## Sum Sq Mean Sq NumDF DenDF F value Pr(>F)
## TIME 219.61 219.61 1 193.65 4.6167 0.0329 *
## Age 1671.56 1671.56 1 231.11 35.1391 1.108e-08 ***
## TIME:Age 26.93 26.93 1 192.19 0.5662 0.4527
## Signif. codes: 0 '***' 0.001 '**' 0.01 '*' 0.05 '.' 0.1 ' ' 1

**Anova**(**lmer**(vlmt6**~**TIME*****Gender**+**(1**|**ES), data = COP_vlmt6_long))

## Type III Analysis of Variance Table with Satterthwaite's method
## Sum Sq Mean Sq NumDF DenDF F value Pr(>F)
## TIME 17.8114 17.8114 1 225.63 5.9465 0.01552 *
## Gender 2.7395 2.7395 1 268.80 0.9146 0.33976
## TIME:Gender 0.2086 0.2086 1 225.63 0.0696 0.79212
## Signif. codes: 0 '***' 0.001 '**' 0.01 '*' 0.05 '.' 0.1 ' ' 1

**Anova**(**lmer**(vlmt6**~**TIME*****Gender**+**(1**|**ES), data = PCBT_vlmt6_long))

## Type III Analysis of Variance Table with Satterthwaite's method
## Sum Sq Mean Sq NumDF DenDF F value Pr(>F)
## TIME 76.351 76.351 1 155.72 24.8964 1.601e-06 ***
## Gender 21.297 21.297 1 191.81 6.9444 0.009096 **
## TIME:Gender 7.348 7.348 1 155.72 2.3961 0.123669
## Signif. codes: 0 '***' 0.001 '**' 0.01 '*' 0.05 '.' 0.1 ' ' 1

**Anova**(**lmer**(vlmt6**~**TIME*****Age**+**(1**|**ES), data = COP_vlmt6_long))

## Type III Analysis of Variance Table with Satterthwaite's method
## Sum Sq Mean Sq NumDF DenDF F value Pr(>F)
## TIME 11.1740 11.1740 1 220.93 3.7932 0.05273 .
## Age 0.2293 0.2293 1 265.92 0.0778 0.78047
## TIME:Age 4.4123 4.4123 1 221.93 1.4978 0.22231
## Signif. codes: 0 '***' 0.001 '**' 0.01 '*' 0.05 '.' 0.1 ' ' 1

**Anova**(**lmer**(vlmt6**~**TIME*****Age**+**(1**|**ES), data = PCBT_vlmt6_long))

## Type III Analysis of Variance Table with Satterthwaite's method
## Sum Sq Mean Sq NumDF DenDF F value Pr(>F)
## TIME 9.707 9.707 1 164.85 3.1102 0.07966 .
## Age 110.080 110.080 1 199.49 35.2711 1.256e-08 ***
## TIME:Age 1.281 1.281 1 163.99 0.4105 0.52264
## Signif. codes: 0 '***' 0.001 '**' 0.01 '*' 0.05 '.' 0.1 ' ' 1
